# Isolated absent pulmonary valve syndrome with intact ventricular septum in a child with CHARGE syndrome: a rare case report and review of the literature

**DOI:** 10.3389/fcvm.2026.1727914

**Published:** 2026-06-18

**Authors:** Azzam Alqashami, Maryam Alkhalaf, Faleh Al Qahtani

**Affiliations:** 1 Department of Pediatrics, College of Medicine, Majma’ah University, Majma’ah, Saudi Arabia; 2 Pediatric Cardiology, King Fahad Medical City, Riyadh, Saudi Arabia; 3 King Abdulaziz Cardiac Center, National Guard Health Affairs, Riyadh, Saudi Arabia; 4 College of Applied Science, King Saud Bin Abdulaziz University for Health Sciences, Riyadh, Saudi Arabia

**Keywords:** absent pulmonary valve, CHARGE syndrome, CHD7 mutation, fetal echocardiography, intact ventricular septum, non-compaction cardiomyopathy, pulmonary valvuloplasty

## Abstract

**Background:**

Absent pulmonary valve syndrome (APVS) is a rare congenital heart defect usually associated with tetralogy of Fallot. Isolated APVS with intact ventricular septum (IVS) is exceptionally uncommon, and no previous link to CHARGE syndrome has been described.

**Case presentation:**

We report a newborn girl diagnosed antenatally with isolated APVS-IVS. Postnatal evaluation revealed dysplastic absent pulmonary valve with free regurgitation, biventricular non-compaction, and multiple extracardiac anomalies including esophageal atresia, right aortic arch with left ductus arteriosus, and central nervous system malformations. Genetic testing identified a heterozygous CHD7 variant consistent with CHARGE syndrome. The patient showed spontaneous improvement in ventricular function after ductal closure and underwent successful balloon pulmonary valvuloplasty at four weeks of age with favorable long-term outcome.

**Conclusion:**

This is the first reported case of isolated APVS-IVS associated with CHARGE syndrome. The observation of biventricular non-compaction and vascular ring raises the possibility of a unique developmental mechanism within the CHARGE spectrum. Early diagnosis and individualized management are key to optimizing outcomes.

## Introduction

**Absent pulmonary valve syndrome (APVS)** is a rare congenital cardiac malformation most commonly associated with tetralogy of Fallot. The occurrence of APVS with an intact ventricular septum (IVS) is exceptionally uncommon, with only a few cases reported in the literature. Prenatal diagnosis has been described in limited reports. Here, we present a case of isolated APVS-IVS detected antenatally, highlighting its myocardial, cardiac, and extracardiac associations, as well as its clinical course. Genetic testing revealed a pathogenic **CHD7 mutation**, consistent **with CHARGE syndrome**. To our knowledge, this represents the first reported case of isolated APVS-IVS associated with CHARGE syndrome.

The coexistence of **isolated APVS-IVS** with multisystem anomalies represents a diagnostic and management challenge. The identification of a **CHD7 pathogenic variant** in this patient expands the known cardiac phenotypes associated with **CHARGE syndrome**, suggesting a possible shared developmental pathway. Reporting such a case is important to improve understanding of this rare entity, aid in prenatal counseling, and guide multidisciplinary care planning. Moreover, the observed fluctuations in left ventricular function and their relation to ductal patency, as noted in previous reports, provide additional insights into the hemodynamic behavior and natural course of isolated APVS-IVS.

## Case report

A female neonate was delivered at 37 weeks of gestation following a fetal echocardiographic diagnosis of isolated absent pulmonary valve syndrome with intact ventricular septum (APVS-IVS). The prenatal study demonstrated a small tricuspid valve, a thickened right ventricle (RV) with depressed systolic function, and a vascular ring composed of a right aortic arch with left ductus arteriosus and bidirectional flow across the ductus arteriosus. No evidence of fetal hydrops was observed.

The parents were non-consanguineous but belonged to the same tribe and had two healthy sons. The mother's obstetric history included one first-trimester miscarriage and one neonatal death attributed to nuchal cord strangulation.

The neonate was vigorous at birth with a good Apgar score, but her oxygen saturation did not exceed 85%, prompting admission to the neonatal intensive care unit (NICU) for evaluation. She was awake, active, and mildly dysmorphic, with no facial asymmetry, a high-arched intact palate, no ear tags or sinuses, and a normal head and neck examination. Cardiovascular assessment showed good perfusion, a single second heart sound, and a To-and-fro murmur at the left upper sternal border, with mild respiratory distress. The abdomen was soft, bowel function was normal, and a single umbilical artery was identified.

**Figure 1:**
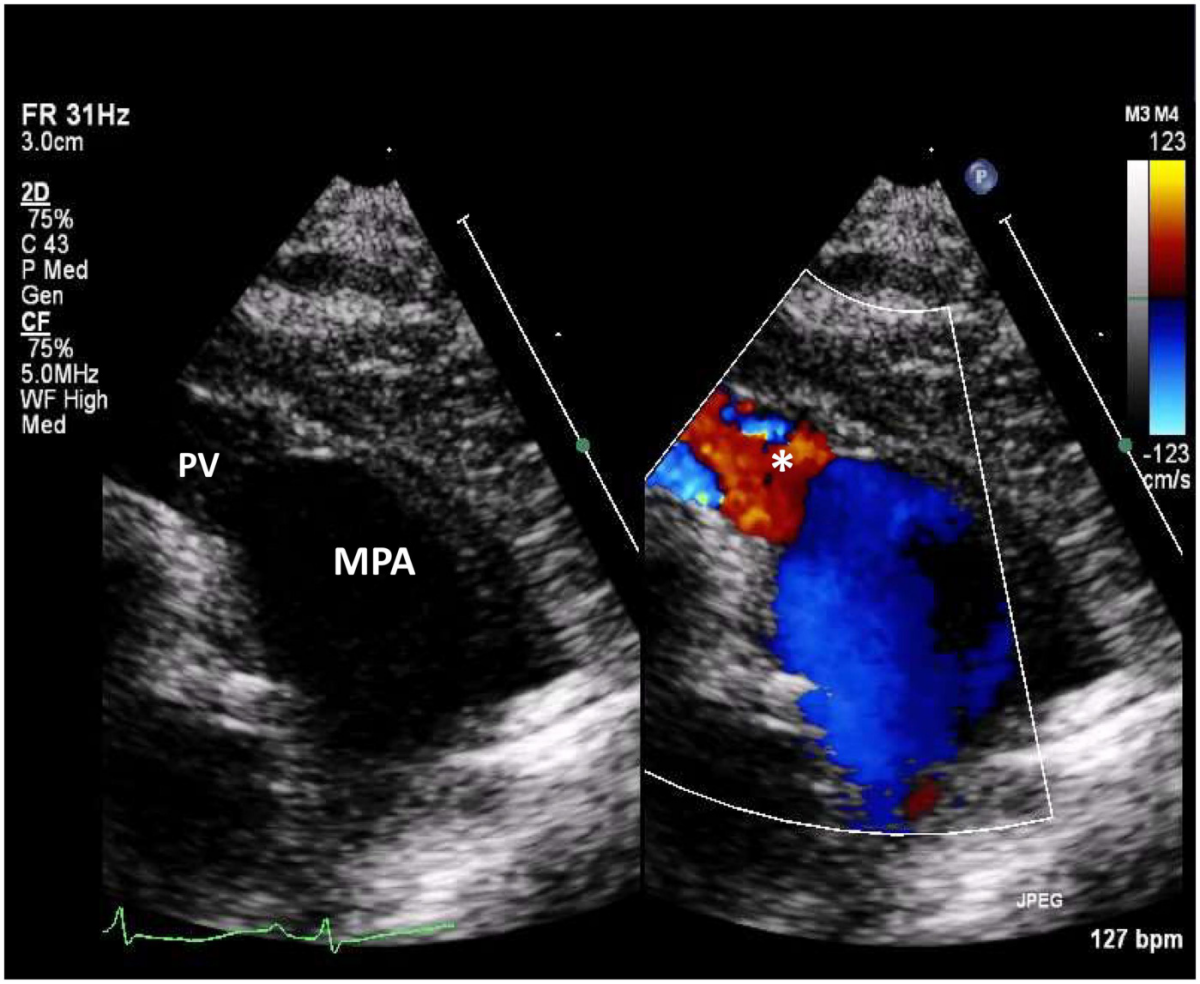
Transthoracic echocardiography, parasternal long axis view showing absent pulmonary valve with stenosis at rudimentary valve area, post stenotic dilatation and pulmonary regurgitation. PV, Pulmonary valve; MPA, main pulmonary artery; and asterisk (*) demonstrate free pulmonary regurgitation.

Transthoracic echocardiography confirmed isolated APVS-IVS with a small tricuspid valve (7 mm, *Z* = −2.4), thickened right ventricle and moderately depressed function, and a narrow pulmonary annulus (4 mm, *Z* = −4.5) showing post-stenotic dilatation and free regurgitation (Figure 1). A vascular ring was present, formed by a right aortic arch, aberrant left subclavian artery, and left ductus arteriosus with left-to-right shunt. Both ventricles showed hypertrophy with non-compaction features and mildly reduced LV systolic function.

Chest x-ray demonstrated a coiled orogastric tube with gas in the stomach, consistent with esophageal atresia. At 5 days of age, the patient underwent exploratory thoracotomy, confirming esophageal atresia with tracheoesophageal fistula, which was successfully repaired. Ultrasound and brain MRI revealed absent septum pellucidum, ventriculomegaly, thinning and dysgenesis of the corpus callosum, mild intraventricular hemorrhage, and mild hypoxic-ischemic injury. Renal ultrasound showed bilateral grade I hydronephrosis.

The postoperative course was uneventful, except for transient hemodynamic instability during repair of the esophageal atresia, requiring prostaglandin infusion for two days. Subsequently, the patient received supportive management in the NICU. Serial echocardiography showed progressive left ventricular (LV) dysfunction over the first three weeks of life, which improved spontaneously without heart failure therapy. As LV function recovered, the peak gradient across the pulmonary valve increased to 90 mmHg, prompting balloon pulmonary valvuloplasty at four weeks of age.

**Figure 2:**
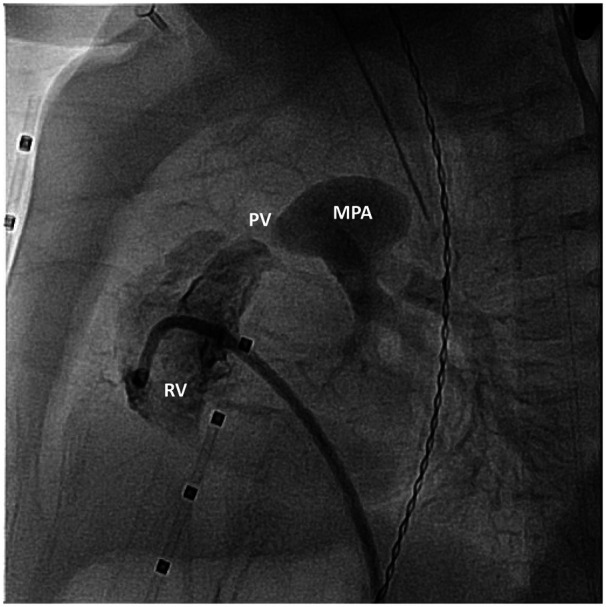
RV angiogram, lateral view, showing stenosis at pulmonary valve area with post stenotic dilatation and good size pulmonary artery branches. RV, right ventricle; PV, pulmonary valve; and MPA, main pulmonary artery.

Angiography revealed a dysplastic absent pulmonary valve (diameter 5 mm). A 6 × 20 mm SABER balloon was used for pulmonary valvuloplasty, resulting in improved forward flow with free pulmonary regurgitation, and good-sized pulmonary artery branches, but an abnormal hypoplastic deep branching pattern (Figure 2). The peak-to-peak gradient decreased from 50 to 35 mmHg, and RV systolic pressure dropped from supra-systemic pressure to 70% of systemic pressure. Follow-up echocardiography confirmed sustained improvement in pulmonary forward flow and LV and RV function, and the patient was discharged at 6 weeks in good condition on room air and full oral feeds.

**Figure 3:**
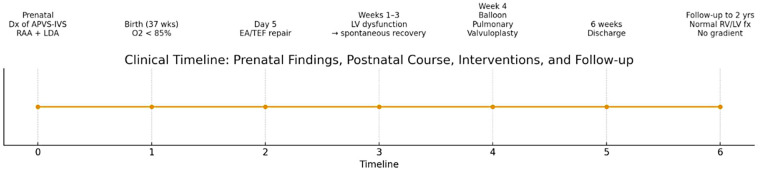
Timeline summarizing the clinical course of the patient, including prenatal diagnosis of isolated absent pulmonary valve syndrome with intact ventricular septum (APVS-IVS), early postnatal findings, surgical repair of esophageal atresia/tracheoesophageal fistula, evolution of ventricular function, balloon pulmonary valvuloplasty at four weeks of age, and subsequent follow-up demonstrating normalization of biventricular function with sustained improvement up to two years of age.

Genetic testing revealed a normal karyotype (46, XX) and a heterozygous likely pathogenic variant in the *CHD7* gene, confirming a diagnosis of autosomal dominant CHARGE syndrome. During follow-up, the patient demonstrated an excellent clinical course along with right ventricular growth, good pulmonary forward flow, and normal biventricular function. Serial studies up to two years of age revealed no significant gradient across the pulmonary valve and sustained normal ventricular function (Figure 3).

## Discussion

The present case was diagnosed antenatally with isolated absent pulmonary valve syndrome with intact ventricular septum (APVS-IVS). Postnatal evaluation revealed multiple congenital anomalies, and genetic testing confirmed CHARGE syndrome due to a *CHD7* mutation. The cardiac findings included a left ductus arteriosus, right aortic arch with aberrant left subclavian artery forming a complete vascular ring, and biventricular non-compaction. Extracardiac features involved a single umbilical artery, as well as central nervous system, urogenital, and gastrointestinal anomalies.

### Anatomical variants and rare associations of APVS

Absent pulmonary valve syndrome (APVS) is a rare congenital heart defect, most often linked to tetralogy of Fallot (TOF) (1). It can also occur, though rarely, with an intact ventricular septum (IVS) (2). Three subtypes are recognized: APVS with TOF, isolated APVS-IVS, and APVS with tricuspid atresia; the first accounts for >80% of cases (3). In a review of 71 patients, Axt-Fliedner et al. found 83% associated with TOF and 17% with isolated APVS-IVS (1). Other rare associations include tricuspid atresia, Ebstein's anomaly, and absence of the aortic valve (1, 2). The ductus arteriosus is typically absent in isolated APVS-IVS (1, 2); however, our patient had a patent ductus arteriosus, a rare feature that could modify postnatal hemodynamics.

Furthermore, in the largest review of APVS cases, while a right aortic arch (RAA) has been reported only in TOF-associated APVS, isolated APVS-IVS cases usually have a left aortic arch (1). In contrast, our patient showed a RAA with a left ductus arteriosus and aberrant left subclavian artery, forming a complete vascular ring. This mirror-image configuration appears to be unique among reported isolated APVS-IVS cases.

### Prenatal diagnosis and hemodynamic considerations

Prenatal diagnosis of isolated APVS-IVS has been described only in a few reports. Rakha et al. reviewed cases up to 2020 and identified 30 fetally diagnosed patients (2). In our case, the defect was detected early in fetal life, allowing timely counselling and close monitoring. Axt-Fliedner et al. observed hydrops fetalis in 69% of similar cases (1), whereas no hydrops developed in our patient.

Early heart failure may occur in isolated APVS-IVS with a large patent ductus arteriosus, occasionally necessitating early closure (2). In our patient, LV systolic function improved spontaneously after ductal closure, despite biventricular non-compaction.

### Biventricular non-compaction

Interestingly, this case demonstrated non-compaction of both the left and right ventricles. A similar finding was reported by Axt-Fliedner et al., who described a case of isolated APVS-IVS associated with membranous tricuspid atresia and right ventricular non-compaction (1). Reviewing the literature and the family history of our patient revealed no alternative explanation for the biventricular non-compaction, including any link to the CHD7 gene. This observation raises the possibility of a distinct developmental relationship between non-compaction cardiomyopathy and isolated APVS-IVS, which warrants further investigation.

### Clinical outcome and follow-up

The outcome and optimal management of isolated APVS-IVS have rarely been discussed. Our patient initially responded to conservative management, but later required balloon pulmonary valvuloplasty for an increasing gradient across the pulmonary valve. LV function improved markedly after spontaneous ductal closure, without the need for heart failure medications. Both short- and long-term follow-up showed favorable progress, with no symptoms, progressive RV growth, and improved pulmonary forward flow and decreasing valve gradient on serial echocardiography.

### Genetic associations and CHARGE syndrome

Extracardiac anomalies are frequently observed across APVS subtypes, mainly involving the central nervous system (CNS), urogenital system (GU), and gastrointestinal tract (GIT) (1). Our patient exhibited CNS, GIT, and GU anomalies, as previously described.

APVS with TOF has been linked to 22q11.2 microdeletion, while other APVS variants have not shown strong chromosomal associations (4). Two isolated APVS-IVS cases with trisomy 18 have been reported (1).

CHARGE syndrome is characterized by coloboma, heart defects, choanal atresia, growth and developmental delay, genital anomalies, and ear abnormalities or deafness (5). Some patients have CHD7 mutations on 8q12, as in our case (6). In a review of 197 patients with CHARGE syndrome, cardiac defects included conotruncal anomalies (32%), TOF (26%), AVSD (19%), PDA (17%), outflow anomalies (14%), aortic arch malformations (6%), ASD (6%), and VSD (3%) (7). To the best of our knowledge, no previous reports have documented an association between CHARGE syndrome and isolated APVS-IVS, making this the first reported case of such a combination.

## Conclusion

Isolated APVS-IVS is a rare entity distinct from TOF with absent pulmonary valve. Although not previously linked to specific genetic syndromes, we report a pathogenic CHD7 variant consistent with autosomal dominant CHARGE syndrome, which is the first such association described. The coexistence of a patent ductus arteriosus may transiently impair LV function, which improves after closure. We also suggest a possible link between APVS-IVS and ventricular non-compaction. Management should be individualized; pulmonary valvuloplasty proved effective in this case.

## Data Availability

The original contributions presented in the study are included in the article/Supplementary Material, further inquiries can be directed to the corresponding author.
